# Impact of different farming scenarios on key soil sustainability indicators driving soil carbon and system productivity of rice-based cropping systems

**DOI:** 10.3389/fpls.2024.1408515

**Published:** 2024-11-01

**Authors:** Ajay Kumar Mishra, Piyush Kumar Maurya, Sheetal Sharma

**Affiliations:** Sustainable Impact through Rice-Based Systems, International Rice Research Institute South Asia Regional Centre, Varanasi, India

**Keywords:** carbon pools, cover crop, crop yield, environment, natural farming, reduce-tillage, soil health, sustainability

## Abstract

This research explores the relationships among soil characteristics, carbon dynamics, and soil biome in rice-based cropping systems across four farming scenarios: conventional farming, organic farming with conventional tillage, integrated nutrient management, and conservation agriculture with zero tillage. Conducted at the International Rice Research Institute, India (2020-2022), the study analyzed physical, chemical, and biological soil parameters. The findings reveal significant effects of farming scenarios on soil organic carbon (SOC), available nitrogen (N), phosphorus (P), and potassium (K), with no notable impact on bulk density, pH, electrical conductivity, or water-holding capacity. Organic farming enhanced microbial health, showing microbial biomass carbon (MBC) at 194.0 μg g^-1^, microbial biomass nitrogen (MBN) at 134.2 μg g^-1^, and dehydrogenase activity (DHA) at 36.80 μg TPF h^-1^ g^-1^, reflecting a more active microbial community important for nutrient cycling. Conservation agriculture reduced soil compaction, promoting better root growth and water penetration, leading to higher crop yields (10.95 ± 0.49 t ha^-1^). The study highlights the role of SOC in enhancing soil health, nutrient availability, and crop productivity, emphasizing sustainable agricultural practices.

## Graphical Abstract

CF, Conventional farming; CA, Conservation agriculture; INM, Integrated nutrient management, OF, Organic farming along with various soil quality parameters.

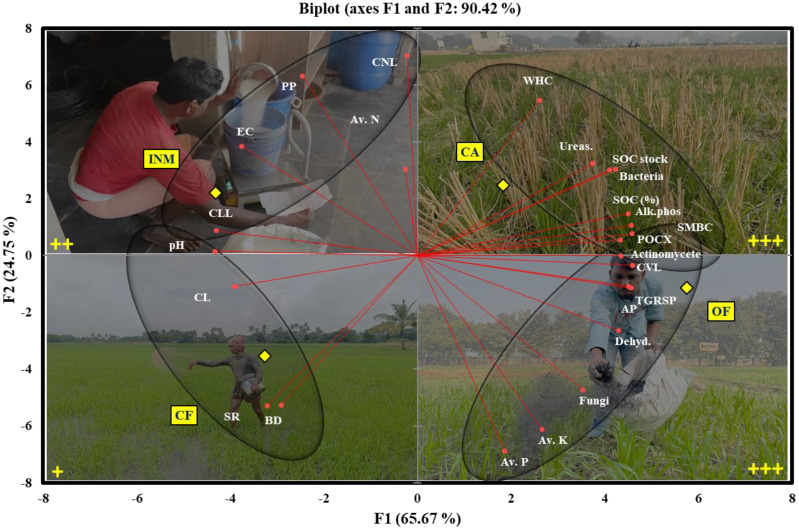

## Highlights

- The study compared four modified farming methods showing varied benefits over the conventional framing.
- Organic farming boosts soil microbial health as compared to conventional farming.
- Conservation agriculture reduces soil compaction, benefiting root growth and enhancing crop yields.
- Both organic farming and conservation agriculture improve soil organic carbon, aiding in carbon sequestration.
- Conservation agriculture achieves the highest crop yield, highlighting the efficacy of sustainable practices.

## 1 Introduction

Climate change and its impact on food production is a pressing global challenge (FAO, 2019). Continuous application of chemical-based fertilizers is another challenge, degrading the soil fertility and causing acidification. Several investigations have found alterations in soil microbial populations after fertilizer application (Wang et al., 2022; Sivojiene et al., 2021). Sivojiene et al. (2021) found that organic fertilizer increases microbial biomass and diversity, while Wang et al. (2022) studied how different fertilization methods affect bacterial communities. Ren et al. (2018, 2020) found that appropriate nitrogen and carbon from organic fertilizer boost soil microbial growth, while excess phosphorus from fertilization lowers microbial community variety (Liu et al., 2022). Applying fertilizers, particularly chemicals, can harm soil characteristics and indirectly affect the microbial community (Cheng et al., 2020). The continuous application of chemical fertilizers can promote soil acidification, which negatively impacts soil structure and fertility (Pahalvi et al., 2021).

Further, soil management practices are crucial, as they directly influence soil health, nutrient availability, and carbon sequestration potential (Six et al., 2002; Lal, 2004). Understanding the interrelationships among soil characteristics, carbon input, carbon pools, and the soil biome is essential for developing effective management strategies that enhance soil fertility, improve crop productivity, and mitigate greenhouse gas emissions (Smith et al., 2016; Hinz et al., 2020).

Mineral and organic fertilizers enhance soil fertility (SOC, nutrient profile) and crop yield, acting as crucial nutrient sources in agricultural soils (Wu et al., 2021; Wang et al., 2017; Kpan et al., 2023).

Global and regional data on SOC stocks are available (Mishra et al., 2009). There have been very limited practical data on the effect of land use patterns on soil carbon and nutrient level at different soil depths under the various land use practices, such as conventional farming, forest land to conventional farming, though numerous studies have explored the effects of different farming practices on soil properties and nutrient dynamics (Wang et al., 2019; Zhang et al., 2020). However, there remains a knowledge gap regarding the specific impacts of these practices on Vertisols, on which rice-based cropping systems are frequently followed (Richards et al., 2015). Vertisols are characterized by high clay content and exhibit unique properties, such as high water-holding capacity and shrink-swell behavior, influencing their response to agricultural management practices (Yadav et al., 2017). Additionally, vertical soil profile has a significant role in the plant growth and root biomass and thus on the SOC. The climate crisis is undeterred by population growth, and urbanization plaguing agricultural sustainability through unmanaged agricultural practices is also threatening and alarming (Yadav et al., 2021). Kumar et al. (2022) have reported that crop productivity has decreased and soil health has deteriorated as a consequence of agriculture based on tillage; thus, zero tillage and residue management can improve soil health (Kumar et al., 2021; Singh et al., 2018).

Further, Yadav et al. (2021) reported that integrating cowpea live mulch in no-till and reduced tillage systems enhances crop and soil productivity in the eastern Himalayan region of India. These practices have also resulted in increased water productivity and saving technologies in sensitive regions like the Himalayan regions. Long-term application of organic amendments also positively impacts soil nitrogen and carbon in irrigated semi-arid cropping systems of India.

Globally, the agricultural system produces approx. 4.6 billion tons of agri-waste residues annually that can be used in the field again (Bentsen et al., 2014). Specifically, Indian agriculture contributes about 500 million tonnes of crop residues each year, with over a quarter of these being burned on farms. This practice of dumping or burning agricultural waste harms soil fertility and impacts environmental quality adversely. Additionally, the intense use of energy could lead to a 6% increase in CO2 emissions by the year 2050 (Gielen et al., 2019). So, developing residue utilization-based farming practices is also crucial under the alarming impact of climate change.

The growing risk of decline in soil health caused by anthropogenic activity is another challenging issue. The nexus between soil degradation, nutrient loss, and GHG emissions under various land use practices is critical to estimate and control. Additionally, in continents such as Asia and Africa, the agricultural sector is predominantly characterized by farmers who are both resource-poor and marginal, holding small parcels of land (Das et al., 2021). These farmers exhibit a constellation of challenges, including limited capital, constrained access to institutional credit, a low capacity for risk absorption, and a reliance on subsistence farming practices typically centered around a single annual crop. Consequently, their ability to generate significant marketable surpluses is challenged. Despite these impediments, there is a pressing imperative for these farmers to enhance agricultural productivity per unit of land, water, nutrients, and energy input (Babu et al., 2023).

Thus, the present study was conducted at the International Rice Research Institute, South Asia Regional Centre in Varanasi, Uttar Pradesh, India, to address these research gaps. The study aimed to investigate the influence of four farming scenarios, namely conventional farming, organic farming with conventional tillage, integrated nutrient management, and conservation agriculture with zero tillage, respectively, on soil characteristics, carbon input, carbon pools, and the soil biome in Vertisols under rice-based cropping systems.

Therefore, considering these aspects, comprehensive analyses were performed on the soil’s physical, chemical, and biological parameters. Soil samples were collected before and after crop harvest, and a range of measurements, including soil organic carbon content, available nutrient content, microbial biomass and activity, enzyme activities, microbial population abundances, soil compaction, and distribution of different organic carbon fractions, were conducted. Moreover, understanding the intricate relationships among soil characteristics, carbon input, carbon pools, and the soil biome is crucial for devising evidence-based strategies that promote soil health, enhance nutrient cycling, and mitigate climate change impacts (Mishra et al., 2018; Lal, 2020).

## 2 Materials and methods

### 2.1 The experimental site

**Figure 1:**
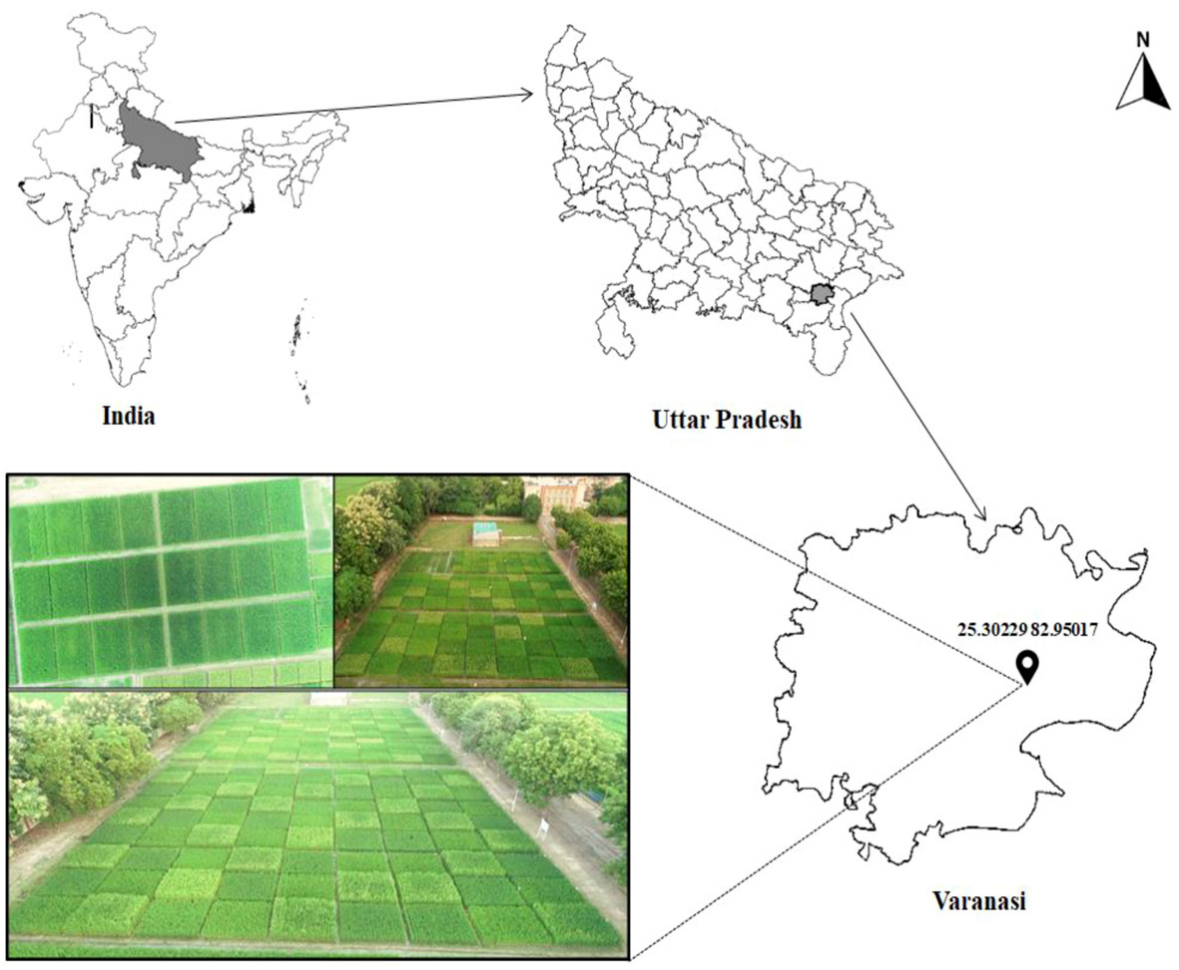
Location of study sites. Comparable to Conventional farming (CF), Organic farming (OF), Integrated nutrient management (INM) and Conservation agriculture (CA) farming systems.

**Figure 2:**
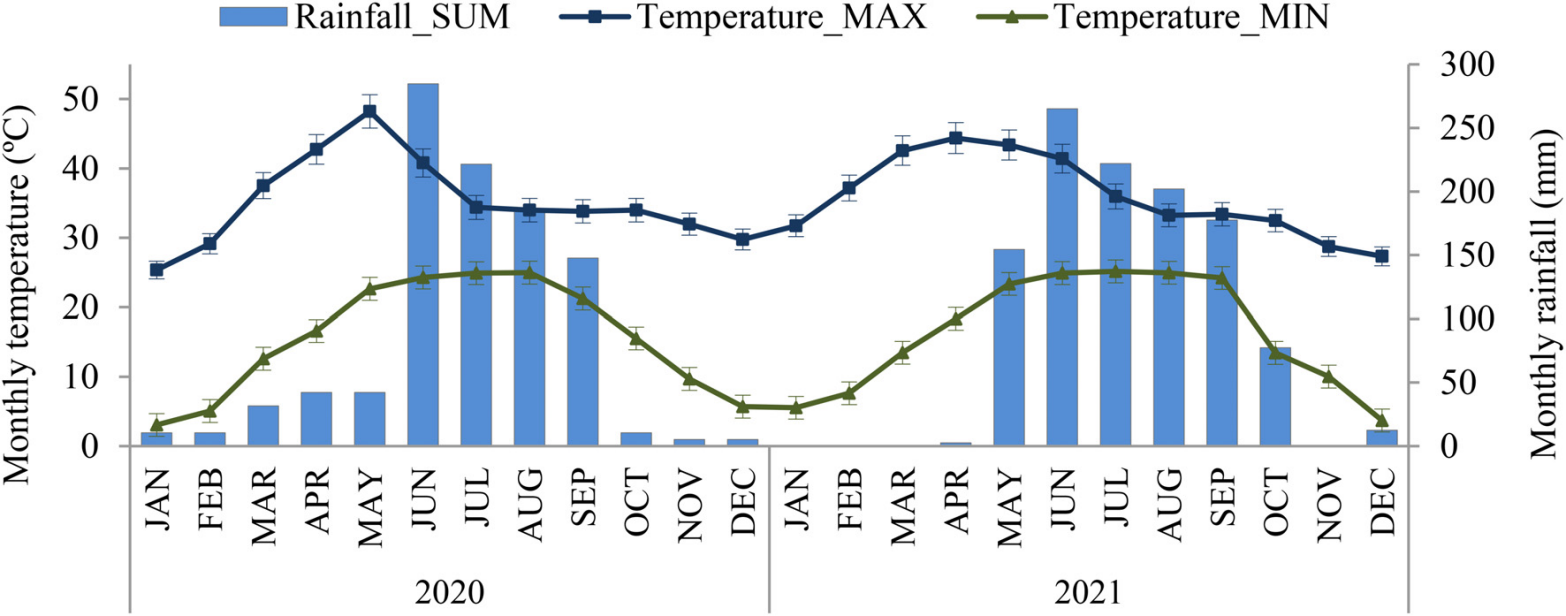
Monthly average maximum-minimum temperature and rainfall distribution for 2020 and 2021.

The experiment was conducted during the year 2020 to 2022 at the International Rice Research Institute South Asia Regional Centre (ISARC), Varanasi (25.30° N – 82.95° E, 83 m above mean sea level), Uttar Pradesh, India (**Figure 1**) with rice-based cropping systems under different farming scenarios. The mean minimum and maximum temperatures at the experimental site during experimental period was 15.5°C and 36°C, respectively. Annual rainfall recorded ranges between 900 and 1113 mm and more than 70% rainfall occurred during the monsoon season from July to September (**Figure 2**). The soil at the experimental site is sandy clay loam and it was classified as *Typic Natrustalf* (US Taxonomy).

The ISARC, Varanasi was selected as it is a site representative of rice-based cropping systems in similar agroecological zones. While the findings provide valuable insights, the results may be limited in their generalizability to other regions with different environmental and soil conditions. We advocate that future research should be conducted across diverse locations to validate these findings and extend their applicability to broader contexts.

### 2.2 Treatments, experimental design and crop management

The experiment was established in a randomized complete block design in which four farming scenarios constituted the treatments as follows: Conventional farming (Scenario 1) ploughing and use of chemical-based fertilizers and pesticides, Organic farming (using bio-pesticide, farm yard manure and compost) with conventional tillage (CT) for rice and wheat (Scenario 2), Integrated nutrient management (Organic and chemical based combined input, Scenario 3), and Conservation agriculture with zero tillage (ZT) for rice and wheat (Scenario 4). There were three replications of each treatment. While rice and wheat were grown under all the scenarios, in Scenarios 2 and 4, ZT mungbean was grown as a Zaid crop between wheat and rice.

The management practices, viz. tillage, sowing method, fertilizer application, water management, crop geometry and spacing (between the rice, wheat and mungbean system, as provided in **Table 1**. It is also same in all the scenarios, 22.5– 22.5– 45 cm), and crop varieties used for growing rice, wheat, and maize under different farming scenarios are described in **Tables 1**, **2**. Crop geometry in agronomy refers to the arrangement of the crop plants in each plots, including the shape of the space available for each plant, and the spacing between rows and plants. While in Scenario 1 wheat was sown by broadcasting the seeds and rice was manually transplanted, in Scenario 3 Happy Seeder machine was used for seeding both rice and wheat.

**Table 1:** Tillage, seed rate, cropping system, and agronomic management practices followed under the four farming scenarios.

Management practices	Scenario 1 (CF^*^)	Scenario 2 (OF^*^)	Scenario 3 (INM^*^)	Scenario 4 (CA^*^)
**Field Preparation**	Rice: CT Wheat: CT	Rice: CT Wheat: RT Mungbean: ZT	Rice: ZT Wheat: ZT	Rice: ZT Wheat: ZT Mungbean: ZT
**Seed rate (kg ha^-1^)**	Rice: 25 Wheat: 100 -	Rice: 25 Wheat: 100 Mungbean: 20	Rice: 25 Wheat: 100	Rice: 25 Wheat: 100 Mungbean: 20
**Sowing method**	Manual transplanting of rice and broadcasting of wheat	Manual transplanting in rice and seed drill sowing in wheat and mungbean	Seeding with a happy seeder machine	Manual transplanting in rice and seed drill sowing in wheat and mungbean
**Crop geometry & spacing (cm)**	Random	(22.5– 22.5– 45 cm)	(22.5– 22.5– 45 cm)	(22.5– 22.5– 45 cm)
**Fertilizer (Nitrogen-Phosphorus and Potassium; NPK) in kg ha^-1^**	Rice: 120:60:40	Nutrients applied through bio-inputs^**^ (**Table 1A**)	75% RDF (90:45:30 NPK) + 25% through bio-inputs^**^ (in the form of bioliquids: This bio-liquid is a new formulation of ISARC and Grassroot Energies Pvt. Ltd, which contains 1% of nitrogen, 0.2% of P and 0.5% of K). Quantity: 200 L/ha, time of application: Basal (0-15), 30-40 DAS and 60-70 DAS)	Nutrients applied through SSNM + 25% of bio-inputs^**^ (basal dose FYM @ 3 t/ha)
**Water management** ***(no. of Irrigation)***	Rice: Soil was wet for up to 20 days after sowing Irrigation applied at hair-line cracks (30-35 irrigations). Wheat: 3-4	Rice: Soil was wet for up to 20 days after sowing Irrigation applied at hair-line cracks (25-30 irrigations) Wheat: 3-4 Mungbean: 1-2	Rice: After transplanting, Irrigation was done by alternate wet and dry methods (20-25 irrigation) Wheat: 3-4	Rice: After transplanting, Irrigation was done by alternate wet and dry methods (20-25 irrigation) Wheat: 3-4 Mungbean: 1-2
**Crop Varieties**	Rice: Arize 6444 Gold Wheat: PBW 187	Rice: Arize 6444 Gold Wheat: PBW 187 Mungbean: Virat	Rice: Arize 6444 Gold Wheat: PBW 187	Rice: Arize 6444 Gold Wheat: PBW 187 Mungbean: Virat

^*^CF, Conventional Farming; OF, Organic farming; INM, Integrated nutrient management; CA, Conservation agriculture practices; RDF, Recommended dose of fertilizer; CT, Conventional tillage; RT, Reduce tillage; ZT, Zero tillage practices.

^**^Bio-inputs OF- Azolla*, BGA*, Vermicompost, Vermiwash and Mycorrhiza.

**Table 1A:** Seed treatments and nutrients applied through bio-inputs.

Growth stage	DAS	Bio-inputs					
		Trichoderma (g/kg seed)	Azolla* (t/ha)	BGA: Blue-green algae* (kg/ha)	Vermicompost (t/ha)	Vermiwash (L/ha)	Mycorrhiza (kg/ha)
Seed treatment	–	10	–	–	–	–	–
Basal	0-15	–	1	15	3	200	10
Active tillering	30-40	–	–	–	3	200	–
Panicle initiation	60-70	–	–	–	3	200	–

DAS, Days after sowing, (*applicable only for rice crop).

**Table 2:** Crop establishment method, cropping pattern and residue management under the four farming scenarios.

Scenarios	Treatments	Crop Rotation	Tillage	Crop establishment method	Residue Management	Water management
**Scenario 1**	CF^*^	Rice-wheat-fallow	Rice: CT Wheat: CT	Rice: Puddled transplanted rice (PTR) with random geometry Wheat: Conventional till (CT) with broadcast seedling	All crop residue removed	Border irrigation
**Scenario 2**	OF^*^	Rice-wheat-mungbean	Rice: CT Wheat: RT Mungbean: ZT	Rice: Puddled transplanted rice (PTR) with random geometry Wheat: Reduce tillage (RT) - line seedling Mungbean: Zero tillage (ZT) with row geometry	Rice & Wheat: 30% anchored residue retained Mungbean: Fully incorporated	Border irrigation
**Scenario 3**	INM^*^	Rice-wheat-fallow	Rice: ZT Wheat: ZT	Rice: Puddled transplanted rice (PTR) with random geometry Wheat: Zero tillage (ZT) - drill seedling	All crop residue incorporated (30% of the above ground biomass- only straw)	Alternate wet drying (AWD)
**Scenario 4**	CA^*^	Rice-wheat-mungbean	Rice: ZT Wheat: ZT Mungbean: ZT	Rice: Puddled transplanted rice (PTR) with random geometry Wheat: Zero tillage (ZT) - drill seedling Mungbean: Zero tillage (ZT) - drill seedling	All crop residue incorporated (30%)	Alternate wet drying (AWD)

^*^CF, Conventional Farming; OF, Organic farming; INM, Integrated nutrient management; CA, Conservation agriculture practices; CT, Conventional tillage; RT, Reduce tillage; ZT, Zero tillage practices.

Fertilizer management practices for different crops under the four scenarios were: Scenario 1 – 120 kg Nitrogen (N), 60 kg Phosphorus (P) and 40 kg Pottassium (K) per ha for rice and wheat, Scenario 2 - bio-inputs (BGA, Azolla, Vermivash, Vermicompost, FYM) for both rice (BGA and Azolla only applicable for paddy) and wheat, and Scenario 3 using 75% of RDF along with 25% bio-inputs (bio-liquid formulations: details mentioned in **Table 1**) and Scenario 4 - Site-Specific Nutrient Management (SSNM) and 25% bio-inputs (bio-liquids formulations). The specific quantities for SSNM are detailed in **Tables 1**, **2**. For SSNM, 75% RDF (90:45:30 NPK) with 25% from bio-inputs was used. RDF in Scenario 1 is the same as specified for other scenarios, except when combined with bio-inputs.

Water management practices also varied, with Scenarios 1 and 2 using frequent irrigations for rice and wheat and Scenario 3 and 4 using alternate wet and dry (AWD) methods. The exact number of irrigations for each scenario is as follows: Scenario 1: 30 irrigations for rice, 3 for wheat; Scenario 2: 26 irrigations for rice, 3 for wheat, 1 for mungbean; Scenarios 3 and 4: 22 irrigations for rice, 3 for wheat, 1 for mungbean.

The crop varieties used in all scenarios were Arize 6444 Gold for rice, PBW 187 for wheat, and Virat for mungbean. These varieties were selected based on their suitability and high yield potential in Eastern Uttar Pradesh, India.

### 2.3 Soil sampling and analysis

After two cropping system cycles (2020-2022), soil samples were collected from the surface layer (0-15 cm) randomly within each plot by using an auger. Three samples within a plot were thoroughly mixed to make composite samples. Composite samples were air-dried under shade. One portion of the sample was ground and the whole amount was filtered using 2.0 mm sieve as per the requirement of different parameter analysis.

The initial soil properties (pH, EC, organic carbon, and available N, P, K) were estimated in air-dried samples using standard protocols (**Table 3**).

**Table 3:** Initial soil properties of the experiment site before commencement of study.

Properties	Value	Method Used
Sand (%)	58	Particle size analysis
Slit (%)	20	
Clay (%)	22	
Textural class	Sandy clay loam	USDA triangle
Bulk density (g cm^-3^)	1.66 ± 0.03	Blake and Hartge, 1986
pH (1:2.5 soil: water)	7.94 ± 0.03	Jackson, 1967
EC (dS m^-1^)	0.151 ± 1.37	Jackson, 1967
Organic carbon (SOC%)	0.53 ± 0.03	Walkley and Black’s method
Available N (kg ha^-1^)	143.1 ± 3.66	Subbiah and Asija, 1956
Available P (kg ha^-1^)	36.2 ± 3.60	Olsen et al., 1954
Available K (kg ha^-1^)	46.4 ± 2.05	

#### 2.3.1 Soil organic carbon stock calculation

Soil organic carbon (SOC) content of the soils under different treatments was determined, following Walkley and Black’s method. SOC stock was calculated using the following formula given below (Datta et al., 2015).


SOC stock in soil=C content ×BD×Soil depth

Where ‘SOC stock’ is in g ha^-1^, ‘C content’ is given in g-C kg^-1^, ‘BD’ is the bulk density of soil in g cm^-3^ and ‘soil depth’ is in cm.

#### 2.3.2 SOC fractions of different oxidizability

Based on the hypothesis that sulphuric acid dilution generates less heat of dilution, three labile fractions of oxidizable SOC were determined using Wakley and Black’s methods. By lowering sulphuric acid normality, which is 24, 18, and 12 N H_2_SO_4_ equivalent, the amount of C thus calculated permitted apportionment of total organic Carbon into four separate pools, which is Pool 1 (OC oxidisable by 12 N H_2_SO_4_), Pool 2 (Difference in C oxidizable by 18~12 *N* H_2_SO_4_), Pool 3 (Difference in C oxidisable by 24~18 *N* H_2_SO_4_) and Pool 4 (Difference between C_tot_ and C oxidisable by 24 N H_2_SO_4_) (Samal et al., 2017).


$$\text{Active pool}=\Sigma \;\left(\text{OC oxidisable by }12{\text{ N H}}_{2}{\text{SO}}_{4}\right)+\left(\text{Difference in C oxidizable by }18∼12{\text{ N H}}_{2}{\text{SO}}_{4}\right)$$

$$\text{Passive pool}=\Sigma \;\left(\text{Difference in C oxidisable by }24∼18{\;N\text{ H}}_{2}{\text{SO}}_{4}\right)+\left({\text{Difference between C}}_{\text{tot}}\text{ and C oxidisable by }24{\;N\text{ H}}_{2}{\text{SO}}_{4}\right)$$

#### 2.3.3 Soil biological analyses

Fresh soil samples were sieved using a 2-mm sieve and sent to the laboratory to evaluate various soil biological characteristics (SMBC, dehydrogenase activity, alkaline phosphatase activity, soil respiration and microbial count). SMBC was calculated using the chloroform fumigation methods (Vance et al., 1987). Dehydrogenase and alkaline phosphatase activities were estimated as described by Dick et al. (1996).

We used the pour plate method for isolating and counting the viable microorganisms present in the soil, which is added with or before molten agar medium before solidification (Zuberer, 1994). The plate was incubated at 32°C inside an incubator and allowed to grow. Colonies were counted after 3 days. To prevent bacterial growth, a total fungal count was performed on rose bengal agar medium (RBA) treated with streptomycin (Martin, 1950). For five days, the plate was incubated at 30°C. Total actinomycetes were counted on actinomycetes isolation agar (AAI) plates that were treated with nalidixic acid to inhibit fungal growth (HiMedia Manual, 2009). AAI plates were incubated at 28°C for seven days.

### 2.4 Crop harvest and yield estimation

At crop maturity, rice, wheat and mungbean crops were harvested manually from 2×2 m^2^ randomly selected three places from each plot for recoding the grain yield. System productivity was calculated on rice equivalent yield (RQY) basis for wheat and mungbean to express the overall impact of treatments. Grain yield was reported at 15% moisture. System productivity (t ha^-1^) was computed using the following Equation.


$$\text{Rice equivalent yield non rice crop }\left(t\;ha-1\;\right)=\frac{\text{Non rice crop yield }\left(\text{t ha}-1\right)\;\times \;MSP\;of\;non\;rice\;crop\;\left(INR\;q-1\right)}{\text{MSP of rice }\left(\text{INR q}-1\right)}$$

Where, MSP- minimum support price and INR-Indian Rupees.

### 2.5 Statistical analysis and evaluation

The Windows-based XLSTAT software program was used to calculate the mean of the data that was analyzed for each parameter and then processed for analysis of variance (ANOVA) to establish the statistical significance of the consequences of various scenarios. The data were subjected to variance analysis to identify relevant effects, and the means were compared using Tukey’s HSD test as a *post hoc* mean separation test (P 0.05). Bivariate Pearson’s correlation coefficients, regression equations, and PCA were computed for all soil variables, namely pH, EC, SOC, BD, DHA, APA, MBC, fungus, bacteria, actinomycetes, and soil respiration under different farming scenarios.

### 2.6 Ethics declaration

No human based study was performed in this work. Experiments were as per the IRRI mandate.

## 3 Results and discussion

### 3.1 Soil chemical properties in different scenarios

The present study investigated the effect of different scenarios on soil chemical properties. The results showed no significant differences in bulk density (BD), pH, EC, and water holding capacity (WHC) among the four farming scenarios. Growing rice and wheat under different farming scenarios exerted a significant impact on SOC, available N, available P and available K (**Table 4**). The SOC content ranged from 0.49% to 0.59% among the farming scenarios; the highest value was observed in Scenario 2. The SOC stock was the highest in Scenario 4 (13.75 Mg C ha^-1^) and the lowest in Scenario 1 (12.65 Mg C ha^-1^). The variations in SOC content and stock can be attributed to the management practices in each scenario, such as the type and amount of organic inputs, tillage practices, and cropping systems. The available N was the highest in Scenario 1 (144 kg ha^-1^) and the lowest in Scenario 2 (140 kg ha^-1^), while available K was highest in Scenario 2 (56 kg ha^-1^) and lowest in Scenario 3 (38 kg ha^-1^). The available P was highest in Scenario 1 (38.5 kg ha^-1^) and lowest in Scenario 3 (12 kg ha^-1^).

**Table 4:** After two years of the experiment, the effect of soil characteristics in various scenarios of agricultural practices.

Scenarios^*^	BD (g cm^-3^)	pH (1:2.5)	EC (dS m^-1^)	WHC (%)	SOC (%)	SOC stock (Mg C ha^-1^)	Av. N (kg ha^-1^)	Av. K (kg ha^-1^)	Av. P (kg ha^-1^)
Scenario 1	1.73 ± 0.03^a^	7.98 ± 0.03^a^	0.123 ± 0.01^a^	55.0 ± 1.96^a^	0.49 ± 0.02^a^	12.65 ± 0.44^a^	144.0 ± 4.00^a^	53.0 ± 2.5^a^	38.5 ± 1.5^a^
Scenario 2	1.65 ± 0.06^a^	7.83 ± 0.07^a^	0.113 ± 0.01^a^	57.3 ± 1.41^a^	0.59 ± 0.06^a^	14.53 ± 0.06^a^	140.0 ± 8.00^a^	56.0 ± 4.0^a^	38.0 ± 3.0^a^
Scenario 3	1.67 ± 0.02^a^	7.97 ± 0.06^a^	0.143 ± 0.02^a^	57.0 ± 1.73^a^	0.51 ± 0.02^a^	12.72 ± 0.04^a^	142.3 ± 7.25^a^	38.0 ± 1.0^b^	12.0 ± 1.1^c^
Scenario 4	1.66 ± 0.01^a^	7.93 ± 0.06^a^	0.123 ± 0.01^a^	57.0 ± 2.65^a^	0.55 ± 0.06^a^	13.75 ± 0.50^a^	150.7 ± 3.56^a^	45.0 ± 7.0^ab^	18.0 ± 2.0^b^
Pr>F(Model)	0.059	0.834	0.132	0.271	0.039	0.920	0.853	0.001	0.0001
Significant	No	No	No	No	No	No	No	Yes	Yes

^*^Scenario1 - CF (Conventional Farming), Scenario2 - OF (Organic farming), Scenario3 - INM (Integrated nutrient management), Scenario 4 - CA (Conservation agriculture practices); EC, Electric Conductivity; BD, Bulk density; SOC, Soil organic carbon; Av. N, Available nitrogen; Av. K, Available potassium; Av. P, Available phosphorus.

Alphabetical symbols (a, b, c etc.) denoted the significance difference between the data.

The differences in available nutrient content among the scenarios can be due to the differences in the soil organic matter content and the nutrient management practices. The findings of this study are consistent with previous studies (Lal, 2004). Magnitude of SOC stocks at any place is decided by various governing parameters, such as, land use pattern, extent of land degradation, soil type, depth profile and total input of biomass C annually. Therefore, practices that increase SOC content, such as conservation tillage, cover cropping, and organic amendments can enhance soil quality and crop productivity. SOC is one of the most important sustainability parameter of soil health and agricultural sustainability that work at the ecosystem interface (Shelef et al., 2018; Mishra et al., 2022b). It also helps quantitatively in mitigating the net rate of greenhouse gas emissions.

The results suggest that management practices that enhance SOC content could improve soil fertility and nutrient availability. Further studies are needed to investigate the long-term effects of different management practices on soil properties and crop productivity.

### 3.2 Scenario-specific alterations in soil biological properties

#### 3.2.1 Soil microbial properties

Significant differences in microbial biomass carbon (MBC), microbial biomass nitrogen (MBN), dehydrogenase activity (DHA), alkaline phosphatase activity (APA), and urease activity (UA) were observed among the four farming scenarios. values of MBC and MBN ranged from 107.9 to 194.0 and 74.4 to 134.2 μg g^-1^ in dry soil, with the lowest values in Scenario 1 and the highest in Scenario 2. In Scenario 2, SMBC and SMBN were 79.9% and 80.6%, respectively and these were statistically higher than in Scenario 1.

Soil dehydrogenase activity (DHA), alkaline phosphatase activity (APA), and urease activity (UA) in different farming practices are presented in **Table 5**. There were significant differences among farming scenarios for DHA and APA, but UA was similar in different scenarios. The DHA ranging from 21.06 to 36.80 μg TPF g^-1^ h^-1^ was found in the order: Scenario 3< Scenario 1< Scenario 4 ≤ Scenario 2. Compared to Scenario 1, DHA was 22.74% lower in Scenario 3 but 42.35% and 5.02% higher in Scenario 2 and Scenario 4, respectively, from Scenario 1.

**Table 5:** Effect of cropping system and different agriculture practices on soil microbial properties after two years of the experiment.

Scenarios	Microbial biomass carbon (μg g^-1^)	Microbial biomass nitrogen (μg g^-1^)	Dehydrogenase activity (μg TPF h^-1^ g^-1^)	Alkaline phosphatase activity (μg p- soil g^-1^ h^-1^)	Urease activity (mg urea g^-1^ soil h^-1^)
Scenario 1	107.8 ± 5.14^b^	74.30 ± 3.50^b^	25.85 ± 0.88^ab^	7.96 ± 0.62^b^	180.5 ± 2.58^a^
Scenario 2	194.0 ± 23.3^a^	134.2 ± 16.1^a^	36.80 ± 0.37^a^	13.4 ± 4.54^a^	185.5 ± 1.50^a^
Scenario 3	111.1 ± 7.10^b^	76.90 ± 4.90^b^	21.06 ± 0.40^b^	7.87 ± 0.27^b^	180.9 ± 5.51^a^
Scenario 4	159.7 ± 13.8^ab^	116.8 ± 10.1^ab^	27.15 ± 5.29^ab^	11.9 ± 0.63^ab^	187.7 ± 1.43^a^

Where; Scenario1- Conventional farming; Scenario2- Organic farming; Scenario3- Integrated nutrient management; Scenario4- Conservation agriculture practices.

Alphabetical symbols (a, b, c etc.) denoted the significance difference between the data.

The highest values of MBC and MBN were recorded in Scenario 2, which suggests a higher potential for C and N cycling in this scenario compared to the others. Similarly, Scenario 2 also showed the highest values for DHA and APA, the indicators of soil microbial activity and nutrient availability, respectively. These results suggest that Scenario 2 is likely to have a more active and diverse microbial community, which is known to play a critical role in SOM decomposition and nutrient cycling (Li et al., 2019). Microbiomes also play an important role in nutrient acquisition, protection from pathogens, boosting plant immunity, competition, tolerances to abiotic stresses, and phytohormone production. Scenario 1 and Scenario 3 had the lowest MBC, MBN, and DHA values, indicating relatively lower microbial biomass and activity. It may be attributed to soil contaminants or environmental stressors, negatively affecting soil microbial communities (Bastida et al., 2016). Scenario 4, on the other hand, showed intermediate values for all the parameters tested, indicating a moderate level of soil microbial activity and nutrient availability. The similar and high UA values observed in all the scenarios suggest that the soil has a high capacity for nitrogen mineralization, which is essential for plant growth and productivity (Kumar et al., 2019). However, it is worth noting that excessive UA can lead to N leaching and subsequent environmental degradation. Thus, high UA values suggest that precise and site-specific N management practices should be implemented to minimize the negative ecological impacts. Previous studies have reported a negative correlation between soil depth and enzyme activity, specifically with regards to sucrase and urease (Taylor et al., 2002; Xu et al., 2022). The presence of increased levels of oxygen and nutrients (provided by organic/bio inputs) within the topsoil fostered a heightened state of microbial activity, and consequently leading to a greater degree of biochemical reactions occurring within the soil. Studies have consistently demonstrated that the use of nature based, organic, bio-inputs amendments leads to a significant increase in the activity of enzymes such as urease in the soil (Lazcano et al., 2013; Liang et al., 2014). Their finding suggests that soil amendments have a positive impact on the biochemical properties of soil.

The results of this study provide valuable insights into the microbial community structure and soil nutrient dynamics in different scenarios. The high values of MBC, MBN, DHA, and APA observed in Scenario 2 suggest that organic farming has the potential for enhanced soil health and productivity. Further research is needed to determine the underlying factors responsible for the observed differences among the scenarios and to develop effective management strategies for improving soil quality and productivity.

#### 3.2.2 Soil respiration and microbial population (fungi, bacteria and actinomycetes) count

The results listed in **Table 6** show that the total bacterial counts ranged from 11.2 ± 1.3 to 24.3 ± 0.1 × 105 CFU g^-1^ soil, with the highest abundance in Scenario 2, followed by Scenario 4, Scenario 3, and Scenario 1. In contrast, fungi count ranged from 6.70 ± 0.6 to 13.0 ± 1.4 × 104 CFU g^-1^ soil, with the highest abundance observed in Scenario 2, followed by Scenario 4, Scenario 1, and Scenario 3. The actinomycetes count ranged from 6.80 ± 1.5 to 16.7 ± 1.4 × 105 CFU g^-1^ soil, with the highest abundance observed in Scenario 2, followed by Scenario 4, Scenario 3, and Scenario 1. These results suggest that farming scenarios can differently affect soil microbial communities and that some scenarios may be more favorable to certain microbial groups than others.

**Table 6:** Respiration of soil and the count population of fungi, bacteria, and actinomycetes.

Scenarios	Total bacteria (CFU^*^ × 10^5^ g^-1^ soil)	Fungi (CFU × 10^4^ g^-1^ soil)	Actinomycetes (CFU × 10^5^ g^-1^ soil)	Soil respiration (μg g^-1^ d^-1^)
Scenario 1	11.2 ± 1.3^b^	11.0 ± 3.5^a^	6.80 ± 1.5^b^	56.7 ± 1.5^a^
Scenario 2	24.3 ± 0.1^a^	13.0 ± 1.4^a^	16.7 ± 1.4^a^	52.3 ± 1.4^a^
Scenario 3	15.0 ± 3.0^b^	6.70 ± 0.6^a^	8.00 ± 1.0^b^	53.7 ± 1.0^a^
Scenario 4	22.7 ± 8.4^a^	10.0 ± 1.0^a^	10.3 ± 1.5^a^	52.3 ± 1.5^a^

^*^CFU, Colony forming unit; Scenario 1- Conventional farming; Scenario 2- Organic farming; Scenario 3- Integrated nutrient management; Scenario 4- Conservation agriculture practices.

Compared to Scenario 1 the count of bacteria and actinomycetes were 117.9%, 143.9% higher in Scenario 2, 34.3%, 17.1% higher in Scenario 3 and 103%, 51.2% higher in Scenario 4, respectively. In fungi, Scenario 1, Scenario 2 and Scenario 4 showed 65%, 95% and 50% higher population, respectively than Scenario 3. The trend of microbial counts in the four farming scenarios was in the order as listed in **Table 6A**.

**Table 6A:** Effect of different farming practices on soil microbial population and soil respiration in order.

Parameters	Order in four scenarios^*^	Significant
Bacteria	Scenario 2 ≥ Scenario 4 > Scenario 3 > Scenario 1	Yes; p>0.0001
Actinomycete	Scenario 2 > Scenario 4 > Scenario 3 > Scenario 1	Yes; p>0.0001
Fungi	Scenario 2 > Scenario 1 ≥ Scenario 4 > Scenario 3	No; p>0.078
Soil respiration	Scenario 3 > Scenario 2 ≥ Scenario 4 > Scenario 1	No; p>0.229

^*^Scenario 1- Conventional farming; Scenario 2- Organic farming; Scenario 3- Integrated nutrient management; Scenario 4- Conservation agriculture practices.

The soil respiration rates ranged from 52.3 ± 1.4 to 56.7 ± 1.5 μg g^-1^ d^-1^, with no significant differences observed among the farming scenarios. It suggests that different scenarios did not significantly affect the metabolic activity of soil microorganisms. It is important to note that soil respiration is influenced by various factors, such as soil moisture, temperature, and substrate availability, which were not measured in this study. Therefore, further investigation is needed to fully understand the relationship between soil microbial communities and soil respiration in different scenarios.

The findings of the present study are consistent with previous research that has shown the influence of different management practices on soil microbial communities and their activities (Zhang et al., 2020). It is a well-known fact that organic farming is mostly dependent on the soil’s natural microflora, organic biofertilizers and use of bio-pest control chemicals. When nature based biofertilizers (PGPRs) are applied as seed priming bio-agents or soil inoculants, they multiply and participate in nutrient cycling/availability and benefit the crop in many ways. The application of PGPRs, nitrogen fixers, phosphate solubilizers and AMF improves plants’ performance by enhancing nutrient availability, phyto-hormone production and pest-pathogen control (Mohanram and Kumar, 2019; Poonam et al., 2017; Upadhyay et al., 2018). Further research is needed to fully understand these responses’ mechanisms and develop effective management strategies for enhancing soil health and productivity.

### 3.3 Soil penetration and organic carbon fractions of different oxidizability

**Figure 3:**
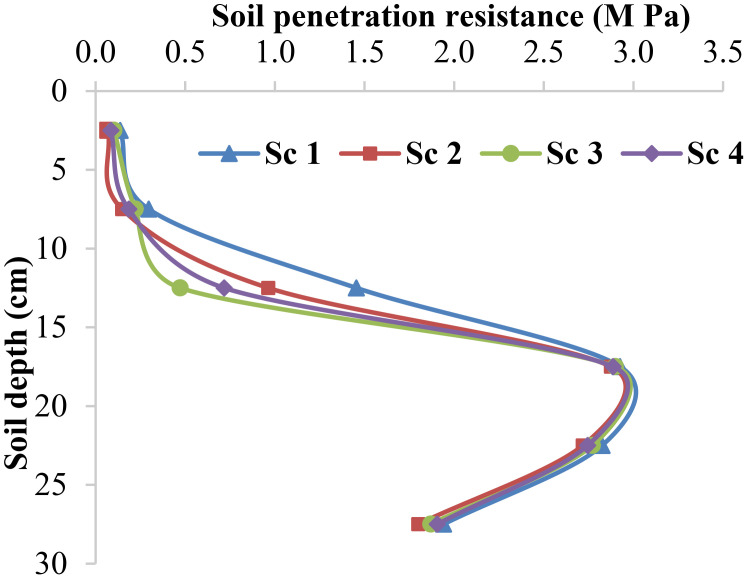
Resistances to soil penetration (M Pa) with depth increase (0-30) under different scenarios.

The measured soil penetration resistance values at different soil depths and scenarios are presented in **Figure 3**. The results indicate variations in soil compaction across the different scenarios and depths. At a soil depth of 5 cm, all scenarios exhibited relatively low penetration resistance values, ranging from 0.07 MPa to 0.14 MPa. This suggests minimal compaction at this shallow depth, which could be attributed to reduced external forces or a less compacted topsoil layer. As the depth increased to 10 cm, the soil penetration resistance values generally doubled across all scenarios. This suggests a gradual increase in soil compaction, potentially due to increased weight-bearing and compaction forces exerted on the underlying soil layers. At a depth of 15 cm, significantly higher soil penetration resistance values were observed compared to the shallower depths, ranging from 0.47 MPa to 1.45 MPa.

The data as plotted in **Figure 3** suggest the presence of compacted soil layers, which can hinder plants’ root growth and nutrient uptake. The soil penetration resistance values at depths of 20 cm, 25 cm, and 30 cm remained relatively constant across all scenarios, with values ranging from 1.80 MPa to 2.93 MPa. These consistently high resistance values indicate considerable soil compaction at these depths, which may limit root penetration and water movement through the soil profile. The variations in soil penetration resistance across the farming scenarios highlight the impact of different agricultural practices or land use patterns on soil compaction. Further investigations are necessary to identify the specific factors contributing to these differences.

**Figure 4:**
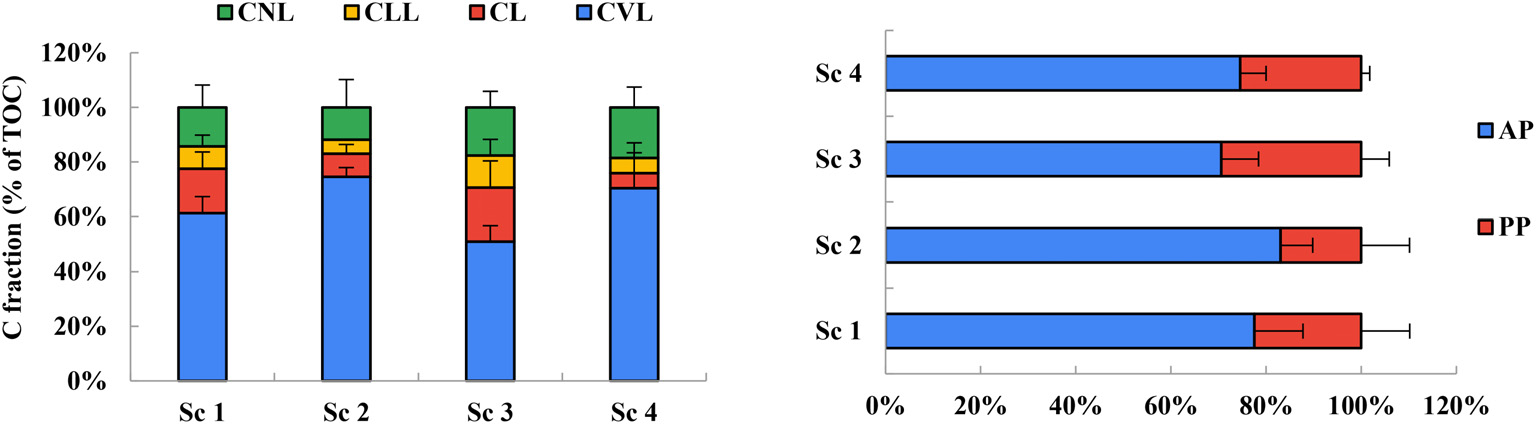
Distribution of soil organic carbon fractions of different lability (as % of total organic C) in different farming practices under rice-based cropping system. (Where; Scenario 1- Conventional farming, Scenario 2- Organic farming, Scenario 3- Integrated nutrient management, Scenario 4- Conservation agriculture practices; CVL- Very Labile; CL- Labile; CLL- Less Labile and CNL- Non-Labile).

Organic farming systems had a (46%) higher pool of very labile C (CVL) compared to conventional (*p*=0.05). Soils under rice–wheat-mungbean cropping systems (Scenario 2 & Scenario 4) exhibited larger very labile C pool than in soils under Scenario 1 & Scenario 3. The results showed that the distribution of SOC fractions varied significantly among the four scenarios. In Scenario 1, the easily oxidizable fraction (CVL) had the highest proportion (0.30), followed by moderately oxidizable (CL, 0.08), slowly oxidizable (CLL, 0.04), and non-oxidizable (CNL, 0.07) fractions. In Scenario 2, the proportion of CVL was the highest (0.44), followed by CNL (0.07), CL (0.05), and CLL (0.03). In Scenario 3, the CVL fraction had the highest proportion (0.26), followed by CNL (0.09), CL (0.10), and CLL (0.06). In Scenario 4, the proportion of CVL was highest (0.38), followed by CNL (0.10), CLL (0.03), and CL (0.03) (**Figure 4**).

This study showed that different management scenarios significantly affected the distribution of SOC fractions in the soil. The organic farming (Scenario 2) had the highest proportion of easily oxidizable SOC, consistent with previous studies showing that organic management practices can increase the amount of labile C in the soil (Chen et al., 2019). This suggests organic management practices can promote SOC turnover and improve soil fertility. The conservation agriculture scenario (Scenario 4) also had a high proportion of easily oxidizable SOC, possibly due to cover crops and reduced tillage, which can enhance SOM (García-Orenes et al., 2013; Mishra et al., 2022b). Although it is generally acknowledged that soil organic matter is primarily concentrated in the top 30 cm of the soil, there is mounting evidence that, despite the concentrations in the subsoil, deeper soil horizons have the ability to sequester significant amounts of SOC. The standard fixed depth of approximately 1 m is typically regarded appropriate in routine soil surveys, which is one factor contributing to the uncertainty. In light of this, it is still unsure how much the worldwide SOC budget has been underestimated.

Further, this research is of relatively short duration (2020-2022). While our research provides important insights into the immediate and short-term effects of various farming practices on soil health and carbon dynamics, these findings may not fully capture the long-term implications. Soil processes, especially those related to carbon sequestration and microbial community shifts, often require extended periods to manifest significant and sustained changes. Future research focused on long-term studies to better understand the enduring effects of these agricultural practices on soil health, carbon storage, and overall ecosystem resilience is needed. This will help to validate the findings presented here, providing a more comprehensive understanding and benefits of sustainable farming practices over time.

### 3.4 Correlation matrix among all soil physicochemical and biological properties

**Figure 5:**
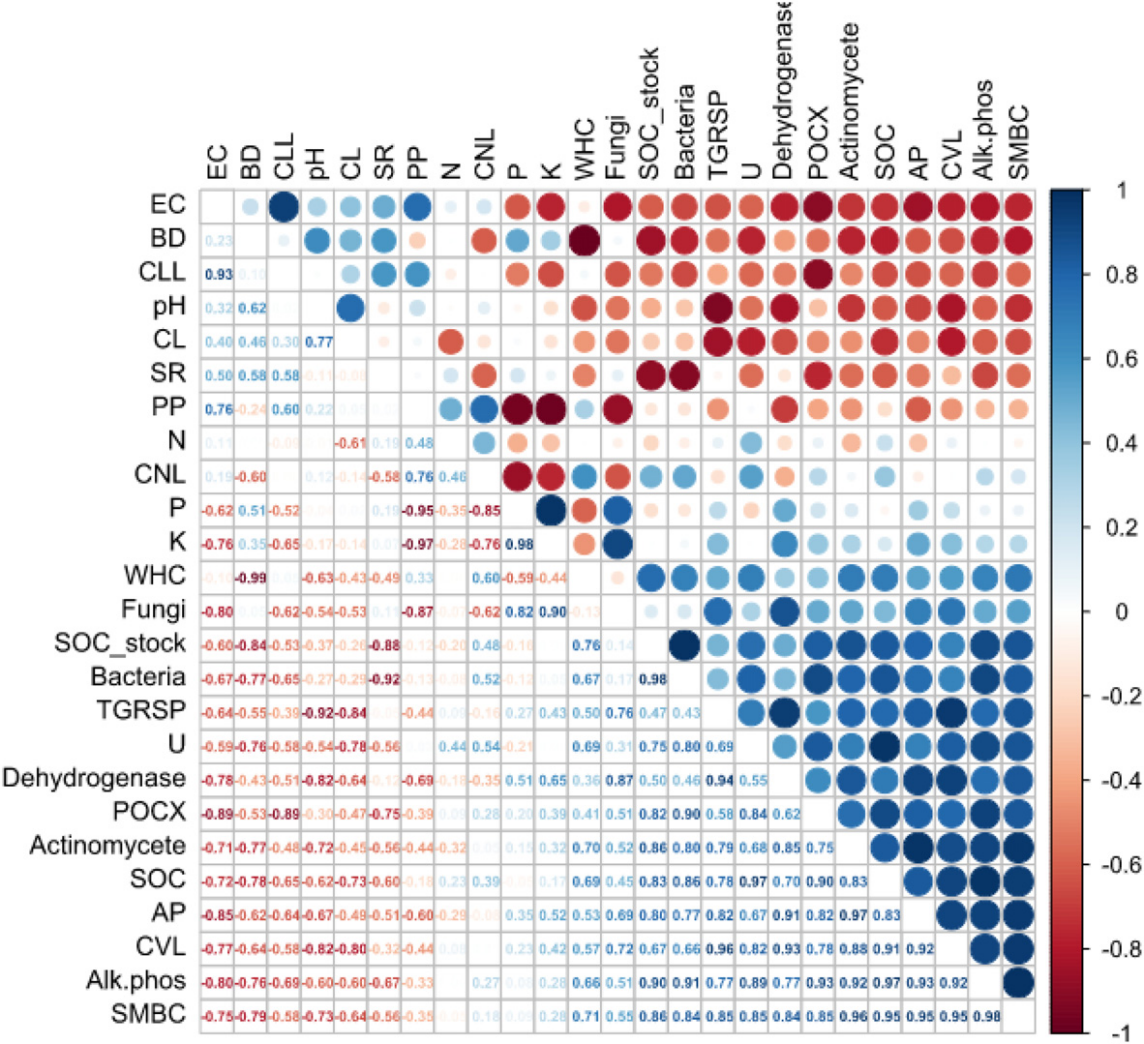
Correlation coefficients (r) among different soil physiochemical and biological properties across all the treatments. (*Values in bold are different from 0 with a significance level alpha ≤ 0.05*) Where; BD, Bulk density; pH, Potential of hydrogen; EC, Electric conductivity; WHC, Water holding capacity; SOC, Soil organic carbon; N, Available nitrogen; K, Available potassium; P, Available phosphorus; SMBC, Soil microbial biomass carbon; Alk.phos, Alkaline phosphatase activity; U, Urease activity; TGRSP, Total glomalin related soil protein; POXC, Permanganate oxidizable carbon; CVL, Carbon very labile; CL, Carbon labile; CLL, Carbon less labile; CNL, Carbon non-labile; AP, Active pool; PP, Passive pool; SR, Soil respiration.

Significant coefficients of correlations (*p* ≤ 0.01 and *p* ≤ 0.05) were observed among most soil physicochemical and biological properties, irrespective of different farming scenarios. Based on the correlation coefficients listed in **Figure 5**, it can be inferred that there are strong correlations between some of the variables.

There is a strong negative correlation (- 0.577) between BD and SOC (%). This suggests that as BD increases, SOC decreases, and vice versa. Similarly, a strong negative correlation exists between SOC and soil pH (- 0.485) indicating that as pH decreases, SOC tends to increase. There is also a strong positive correlation (0.889) between available P (Av. P) and available K (Av. K). This suggests that as the concentration of one nutrient increases, the concentration of the other also tends to increase.

There are strong positive correlations between some soil biological properties, such as bacteria and actinomycetes (r = 0.660), and between bacteria and fungi counts (r = 0.763). These correlations suggest that these microorganisms tend to coexist and influence each other’s populations in the soil. Additionally, there is a strong positive correlation (0.662) between total glomalin-related soil protein (TGRSP) and soil respiration (SR), which suggests that TGRSP may be contributing to soil respiration the ecosystem (**Figure 5**).

### 3.5 Crop yield and rice equivalent system yield

**Table 7** presents the crop yield data from different agricultural practices during the 2020-21 and 2021-22 periods. In the Kharif season of 2020-21, the highest rice yields were obtained from Scenario 3 (4.55 ± 0.20 t ha^-1^), closely followed by Scenario 1 (4.35 ± 0.26 t ha^-1^), while Scenario 2 (2.88 ± 0.21 t ha^-1^) exhibited the lowest yield. Similarly, during the Rabi season, Scenario 3 (3.66 ± 0.19 t ha^-1^) and Scenario 1 (4.03 ± 0.17 t ha^-1^) showed comparable yields, while Scenario 2 (3.00 ± 0.17 t ha^-1^) lagged. When considering the system yield (system productivity), which represents the overall productivity across all seasons, Scenario 4 (10.95 ± 0.49 t ha^-1^) emerged as the most productive approach, significantly outperforming the other scenarios. It was followed by Scenario 1 (8.54 ± 0.44 t ha^-1^) and Scenario 3 (8.35 ± 0.40 t ha^-1^), while Scenario 2 (7.08 ± 0.42 t ha^-1^) exhibited the lowest overall yield.

**Table 7:** In the year 2020-22, pooled yield data (mean ± SD) and system yield (t ha^-1^) as affected by different farming practices followed in four scenarios.

Scenarios^1^ \Season	2020-21				2021-22			
	Kharif	Rabi	Zaid	Rice equivalent system yield^**^ (t ha^-1^)	Kharif	Rabi	Zaid	Rice equivalent system yield^**^ (t ha^-1^)
	Rice yield (t ha^-1^)	Wheat yield (t ha^-1^)	Mung bean yield (t ha^-1^)		Rice yield (t ha^-1^)	Wheat yield (t ha^-1^)	Mung bean yield (t ha^-1^)	
Scenario 1 (CF)^*^	4.35 ± 0.26^a^	4.03 ± 0.17^a^	-	08.54 ± 0.44^a^	5.97 ± 0.36^a^	4.12 ± 0.20^a^	-	10.25 ± 0.57^a^
Scenario 2 (OF)^*^	2.88 ± 0.21^b^	3.00 ± 0.17^b^	0.29 ± 0.01^a^	07.08 ± 0.42^b^	2.98 ± 0.18^b^	3.12 ± 0.16^b^	0.30 ± 0.02^a^	07.33 ± 0.42^b^
Scenario 3 (INM)^*^	4.55 ± 0.20^ab^	3.66 ± 0.19^ab^	–	08.35 ± 0.40^b^	4.54 ± 0.24^ab^	3.68 ± 0.22^ab^	–	08.36 ± 0.47^b^
Scenario 4 (CA)^*^	4.09 ± 0.19^ab^	3.72 ± 0.18^ab^	0.80 ± 0.03^a^	10.95 ± 0.49^a^	4.17 ± 0.21^ab^	4.05 ± 0.17^ab^	0.85 ± 0.04^a^	11.56 ± 0.54^a^

Where, * Scenario 1- Conventional farming; Scenario 2- Organic farming; Scenario 3- Integrated nutrient management; Scenario 4- Conservation agriculture practices.

1 For Scenario details refer to **Table 1**.

Different small letters denote significant differences among the value of mean an individual year, across the scenarios.

(The MSP for rice in 2021–22 was 1940 INR/quintal, mung bean MSP was 7275 INR/quintal, and wheat MSP was 2015 INR/ha).

These results suggest that conservation agriculture, characterized by practices such as reduced tillage and soil cover, can significantly enhance crop productivity (Kumar et al., 2021). The observed variations in crop yields can be attributed to multiple factors, including differences in soil health, nutrient availability, pest management (Mishra et al., 2015; Kumar et al., 2022), and overall management practices associated with each farming approach.

Use of organic fertilizers as opposed to chemical fertilizers can effectively mitigate soil erosion and minimize the potential for aquatic ecotoxicity. Moreover, the implementation of organic farming practices has been found to have a positive impact on soil biodiversity and the presence of both macro and microorganisms. This, in turn, leads to increased income per hectare. However, it is important to note that the yield of crops may be lower when employing OF practices, with a reduction of up to 22%. As per the existing literature, empirical evidence suggests that application of organic fertilizers may result in a decrease in crop yields per hectare. Notably, studies have consistently demonstrated reductions of approximately 20-25% and 50% in yields when comparing OF to conventional fertilizers showed the potential benefits associated with the integration of innovative plant breeding technologies, such as CRISPR/Cas9, within the framework of organic farming practices. Whereas, application of chemical fertilizers has been widely recognized for its positive impact on crop production. However, it is important to acknowledge that the use of chemical fertilizers can also have detrimental effects on soil physicochemical properties and subsequently influence the composition and functioning of soil microbiomes.

### 3.6 Principal component analysis

**Figure 6:**
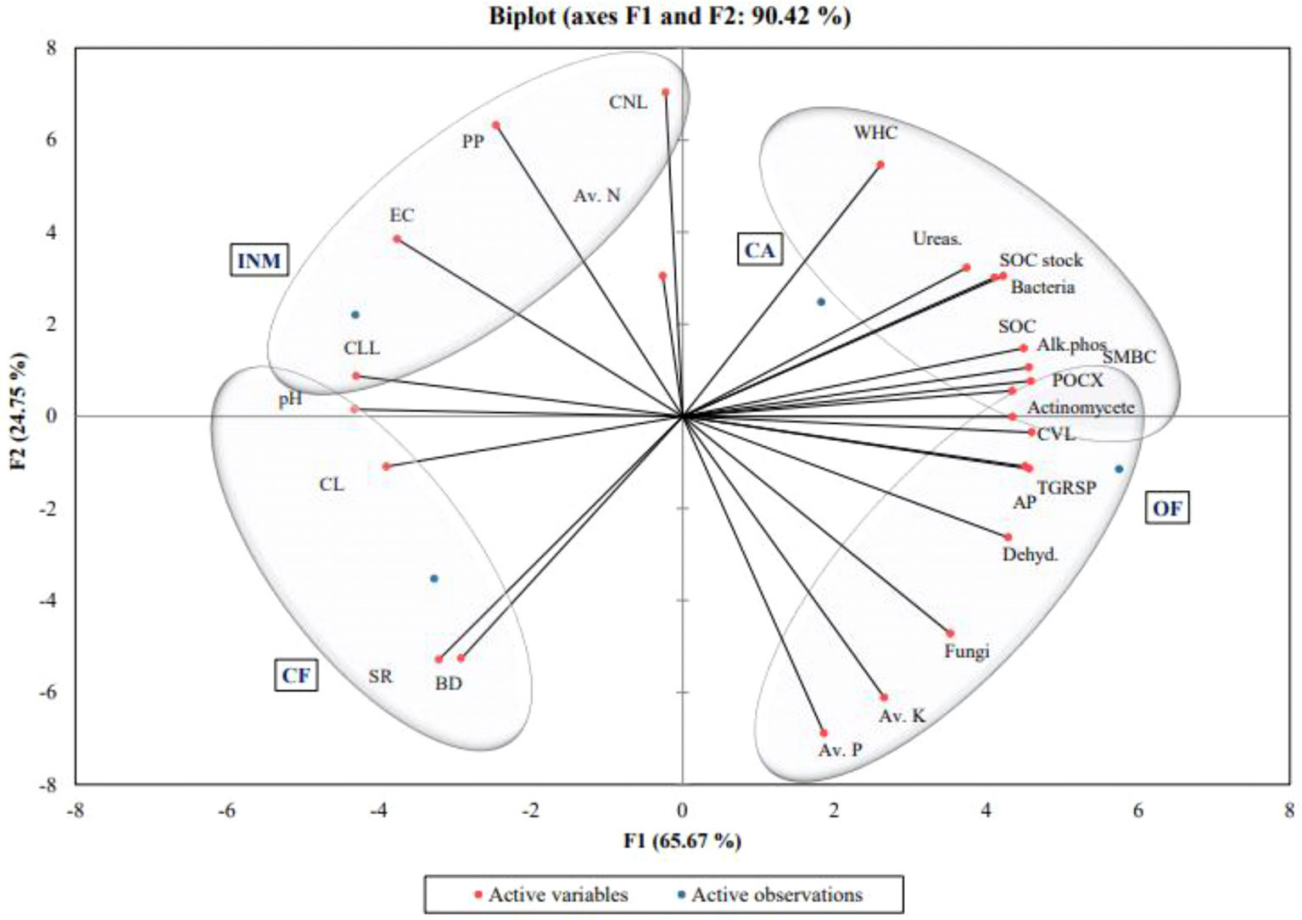
Under various agricultural techniques, the principal component analysis of the soil’s physical-chemical characteristics, enzyme activity, and microbiological parameters.

In the PCA of 21 variables, three principal components were extracted with eigenvalues> 0.9 and these explained 91.18% of the variance. A correlation study (Pearson’s correlation) was performed for all the variables (**Figure 6**). One of the most significant indicators of changes in soil quality was found to be the biomass of soil microbes (Stenberg, 1999). Correlations exist between microbial activity in the soil, microbial biomass, enzyme activity and SOM contents (Chaer et al., 2009). All soil microbial characteristics significantly (p>0.05) correlated with MBC but not with SOM. Soil MBC was found to be a reliable indicator for determining soil quality. DHA was removed from the MDS to reduce redundancy because it is closely connected to MBC. As a result, the MDS continued to use APA.

Based on PCA, most of the microbiome parameters in organic farming were selected to predict the optimum soil quality by different agricultural practices and qualified as key indicators of soil quality. In the present study, all the indicators except BD, available N, pH, (high and low) labile pool of carbon that were retained in the minimum data set were considered good and when in increasing order, these were scored as “more is better”. In contrast, chemical parameters were scored as “less is better”.

## 4 Conclusion and future prospects

The interrelationship between soil characteristics, carbon input, carbon pools, and the soil biome in a Vertisols was found improved in organic and conservation agriculture. Significant effects of the farming scenarios were observed on soil chemical properties, with variations in soil organic carbon, available nitrogen, potassium, and phosphorus. Organic farming exhibited higher soil organic carbon content, while conservation agriculture had the highest soil organic carbon stock. It was also revealed that variations in soil biological properties under organic farming promoted a more active and diverse microbial community. Correlation analysis showed relationships between soil physicochemical and biological properties. The findings highlight the importance of organic farming and conservation agriculture in improving soil quality, nutrient availability, and microbial activity. Conservation agriculture resulted in the highest crop yield, highlighting the efficacy of sustainable practices. As these are the agriculture field based data, findings support for policy development and implementation for large holding farmers for sustainable soil management and production. Further research is needed to understand the underlying mechanisms and develop effective management strategies for enhancing soil health and productivity. To realize the benefits of sustainable production as an integrated strategy, a comprehensive, site-specific framework must be established and scaled.

## Data Availability

The original contributions presented in the study are included in the article/supplementary material. Further inquiries can be directed to the corresponding author.
